# A preliminary investigation into diet adequacy in senior residents of Newfoundland and Labrador, Canada: a cross-sectional study

**DOI:** 10.1186/1471-2458-14-302

**Published:** 2014-04-02

**Authors:** Jing Yan, Lin Liu, Barbara Roebothan, Ann Ryan, Zhi Chen, Yanqing Yi, Peizhong Wang

**Affiliations:** 1 School of Public Health, Tianjin Medical University, Tianjin, China; 2 Division of Community Health and Humanities, Faculty of Medicine, Memorial University of Newfoundland, A1B 3V6 St. John’s, Newfoundland and Labrador, Canada

**Keywords:** Diet adequacy, Senior population, Nutritional epidemiology

## Abstract

**Background:**

Adequate dietary intake is essential to maintain good health. This is particularly true for the elderly. This study investigated the dietary intakes of seniors residing in Newfoundland and Labrador (NL) and assessed the adequacy of nutrients which they consumed as food.

**Methods:**

Between November 2012 and January 2013, we recruited senior residents in NL, aged 65 years or older Participants were required to complete two questionnaires, one food-frequency questionnaire (FFQ) and one general health questionnaire (GHQ). Macro- and micro- nutrients in foods consumed were estimated using the Elizabeth Stewart Hands and Associations (ESHA) nutrient analysis software. The nutrient intakes were compared with appropriate components of the dietary reference intakes (DRIs) adopted by Health Canada to determine adequacy. Various descriptive statistical analyses were performed using SPSS.

**Results:**

One hundred-and-eleven participants (69 females and 42 males) completed the surveys and were included in the analysis. A considerable portion of subjects were overweight (41.7%) or obese (25%), and had at least one chronic illness (86.5%). Many seniors studied did not meet the daily recommendations for dietary intakes of nutrients supported by Health Canada, notably vitamin E (84.7%) and vitamin D (68.5%). Our study also suggests that about 40% of participants consumed more dietary energy as fat than is recommended.

**Conclusion:**

The present study revealed an inadequate consumption of essential nutrients from foods in a noninstitutionalized senior population of NL.

## Background

In Canada, one seventh of the total population was over 65 years of age in 2012 [1].

Due to an increasing life expectancy and a low fertility rate, more than a quarter of Canadians will reach 65 years in 2036 [1]. The elderly are considered at great risk of developing chronic diseases. It has been reported that 89% of senior Canadians had one or more chronic illnesses in 2009 [2]. Thus, as the proportion of the total population represented by seniors increases so will the occurrence of chronic conditions. The consumption of a diet providing adequate and balanced nutrients could improve seniors’ health [1]. The consumption of an adequate diet, supplying energy and nutrients in amounts sufficient to meet needs, should improve nutritional status and enhance disease resistance potentially, prolonging the lifespan for the elderly. For optimal health, seniors should also participate in moderate and regular physical activity and maintain a healthy weight [1]. A healthy diet needs to have a balance of many components such as macronutrients, micronutrients, fluid and energy and at levels adequate to meet but not to exceed human needs. Diets supplying inadequate levels of energy and/or certain nutrients have been associated with decreased cognition and are especially problematic in seniors [3]. Although a large body of scientific literature has provided indisputable evidence that unhealthy diets are associated with many chronic diseases in the elderly population [4-6], little is known about adequacy of the current diet consumed specifically by senior residents of Newfoundland and Labrador (NL), nor how this diet affects their health.

NL is an island in eastern Canada with isolated geography, limited resources, and a unique culture compared with the rest of Canada [7]. Despite the well-known dietary and cultural differences between NL and mainland Canada, there has been limited nutritional epidemiological research conducted among the NL senior population [7]. Dietary intakes of seniors were investigated through Nutrition Newfoundland and Labrador, the NL component of the provincial nutrition surveys, conducted in 1996–97 [8]. Since that time only the Canadian Community Health Survey (CCHS Cycle 2.2) has estimated these intakes [8]. However, the CCHS was not specifically designed for an elderly population and did not have a good representation of the NL senior population. Moreover, CCHS used the 24-hour recall method and was not able to capture seasonal variation of food intake that is particularly relevant to the NL population.

This study is the first nutritional epidemiological study specific to the NL senior population, and thus may provide a current snapshot of the dietary intakes of the older population and some insight into the nutritional status of this group. The purpose of the survey was to describe the dietary intake and health status, and to assess the adequacy of nutrients consumed by the senior population in NL.

## Methods

### Recruitment and study design

Recruitment and data collection were conducted between November 2012 and January 2013. Stratified random digit dialing for household landlines was used to ensure proportional representation of rural/urban residency and gender. First, an initial random sample from the general population was recruited by telephone. After study participants provided verbal consent, a questionnaire package was mailed out, including the Food Frequency Questionnaire (FFQ) and one General Health Questionnaire (GHQ). Participants were required to return the completed questionnaires in a self-addressed and postage paid envelop.

Eligible participants were NL residents aged 65 years or older at the time of the study, noninstitutionalized, had lived in NL for at least two years and were not expected to move out of the province in the next 12 months. Inclusion criteria also required that participants were able to speak and read English at a minimum of a grade 8 level and without apparent cognitive impairment, as information collected from such individuals may not be reliable. All participants who left over 20 continuous items blank on the FFQ and/or who reported energy intakes outside the range of 500–5000 kcal/day were excluded. This matches the exclusionary rules for the food-frequency questionnaire data used by Willett [9].

The FFQ utilized in this study is a modified version of the well-known Hawaii questionnaire [10] and it has been validated for the NL general population [11]. It consists of 175 items organized into 11 food categories and requires participants to recall the number of times each food item was consumed either per day, per week, per month, or rarely/never during the past 12 months. It also requires participants to recall how many months of the year the food was consumed to account for seasonal variation in intake. The GHQ was developed from a Personal Health Questionnaire that was used in previous colorectal cancer studies in NL [12,13] and a Simple Lifestyle Indicator Questionnaire designed for NL residents [14]. The GHQ measured general socio-economic status, lifestyle information, and personal health perception.

### Statistical analysis

Subject characteristics, health-related habits, and chronic health conditions were reported as percentages due to categorical values. According to the International Obesity Task Force (IOTF), a BMI of less than 18.5 is underweight, while a BMI greater than 25 is considered overweight and above 30 is considered obese [15]. A set of three questions in the GHQ focused on physical activity. Light physical activities included light gardening, light housework, leisurely walking, bowling, and playing a musical instrument. Moderate activities were brisk walking, bicycling, skating, swimming, curling, gardening, dancing, Tai Chi or moderate exercise classes. Vigorous physical activities included running, cross-country skiing, lap swimming, aerobics, heavy yard work, weight training, soccer, basketball and other team sports. According to the information from these three questions, subjects were further categorized into four levels: 1) sedentary: light ≤ 3 days per week, 2) less active: light > 3 days per week or moderate ≤ 3 days per week, 3) moderately active: moderate > 3 days per week or vigorous ≤ 3 days per week, and 4) active: vigorous > 3 days per week.

All food items consumed were entered into ESHA Food Processor SQL, version 10.8, nutrient analysis software (ESHA Research Inc., 2010, Salem, Oregon) under the guidance of a professional Registered Dietitian and dietetic graduate students [16]. This software contains more than 35,000 food and beverage items. The nutrient composition data in the ESHA database is compiled from a variety of sources including the USDA Nutrient Database for Standard Reference, the USDA Database for the Continuing Survey of Food Intake by Individuals, the Canadian Nutrient File, manufacturers’ nutrient information, and over 1,000 additional sources of data. Nutrient estimates were calculated using the product-sum method [9,17].

$$\begin{array}{l} \begin{array}{l} \mathrm{Daily}\;\mathrm{nutrient}\;\mathrm{intake}\\ =\;\sum \left[\left(\mathrm{reported}\;\mathrm{consumption}\;\mathrm{frequency}\;\mathrm{of}\;a\;\mathrm{food}\;\mathrm{item},\right)\right)\\ \left(\;\;\mathrm{converted}\;\mathrm{to}\;\mathrm{times}\;\mathrm{per}\;\mathrm{day}\right)\\ \;\times \;\left(\mathrm{portion}\;\mathrm{size}\;\mathrm{consumed}\;\mathrm{of}\;\mathrm{that}\;\mathrm{food}\right)\\ \;\times \left(\mathrm{amount}\;\mathrm{of}\;\mathrm{that}\;\mathrm{nutrient}\;\mathrm{in}\;a\;\mathrm{standard}\right)\\ \left(\left(\;\;\mathrm{serving}\;\mathrm{size}\;\mathrm{of}\;\mathrm{that}\;\mathrm{food}\right)\right] \end{array} \end{array}$$

Briefly, nutrients were classified into two broad categories: macro-nutrients (such as fat and protein) and micronutrients (such as calcium, folic acid, vitamin D and vitamin B12). Initial nutrient intakes were reported as mean, standard deviation (SD) and median. Further, they were compared with the recommended intakes using appropriate components of the Dietary Reference Intake (DRI) [18].

The adequacy of dietary intake was assessed by comparing intakes of a nutrient to the Estimated Average Requirement (EAR) for the age groups 65–70 and >70 years in both genders. The EAR is the mean daily intake value that is estimated to meet the requirement of half the healthy individuals in a life-stage and gender group for that nutrient [19]. Where an EAR was unavailable, as for dietary fibre, the Adequate Intake (AI) was used. Health Canada supports EARs set for micro-nutrients and Acceptable Macronutrient Distribution Ranges (AMDR) for macronutrients. The AMDR is a range of intakes for a particular macronutrient (protein, fat, or carbohydrate), expressed as a percentage of total energy (kcal). Intakes of that nutrient within the AMDR are associated with reduced risk of chronic disease while providing adequate intakes of essential nutrients [18]. Gender comparisons of the prevalence of nutrient inadequacy were examined by using contingency tables.

All analyses were conducted using the Statistical Package for Social Science software version 17.0 (SPSS, Inc., Chicago, IL, USA). The t-test was used to assess difference in nutrient intakes between males and females. Logistic regression analysis was performed for adjusted odds ratio (OR) based on gender, age, marital status, education and energy intake, in order to identify possible associations between demographic factors, inadequate intake of key nutrients and existence of chronic diseases. A *p*-value of <0.05 was considered statistically significant.

### Ethical consideration

This research was approved by the Health Research Ethics Board (HREB) at Memorial University of Newfoundland. (Reference number 12.123)

## Results

### Population characteristics and health status

At the first stage, a total of 1201 phone numbers stratified with rural/urban residency and gender were identified. After screening for eligibility, 252 eligible participants were contacted. Of the 252 eligible, 203 subjects agreed to participate and the survey package was mailed out to them. By March 2013, there were 119 completed packages returned. We excluded a further eight participants according to exclusion criteria and the remaining 111 respondents were involved in the further analysis.

Table 1 presents some socio-demographic characteristics, health-related behaviours and self-reported health status of study participants. The average age was 73.5 years (SD = 64.5, age range: 66–93 years) and 57.8% were older than 70 years. More females (62.2%) than males participated in this study. More than half (54.6%) of the participants received a high school or higher education. The majority of participants were married (75.2%) and had a household income less than $39,999 per year (62.2%). A large proportion of subjects were not current smokers (92.7%), while half (49.5%) of the subjects reported weekly alcohol consumption. Two-thirds of participants presented as sedentary or less active.

**Table 1:** Demographic characteristics of the dietary inadequacy study participants

**Characteristics**	**n**	**%**
**Gender (N = 111)**		
**Male**	42	37.8
**Female**	69	62.2
**Age range (years) (N = 109)**		
**65-70**	46	42.2
**≥70**	63	57.8
**Marital status (N = 109)**		
**Single/divorced/widowed**	27	24.8
**Married/living together**	82	75.2
**Education (N = 108)**		
**No high school certificate**	49	45.4
**High school**	25	23.1
**Post-secondary education**	34	31.5
**Annual household income (N = 111)**		
**≤$39,999**	69	62.2
**$40,000-$99,999**	20	18.0
**≥$100,000**	5	4.5
**Did not disclose**	17	15.3
**Tobacco use (N = 110)**		
**Current smoker**	8	7.3
**Former smoker**	63	57.3
**Never used**	39	35.4
**Alcohol use (N = 109)**		
**Consumes weekly**	54	49.5
**Never used**	55	50.5
**Activity (N = 111)**		
**Sedentary/light activity**	74	66.7
**Active**	32	28.8
**Vigorous**	5	4.5
**BMI (kg/m**^**2**^**) (N = 108)**		
**BMI < 18.5**	2	1.8
**18.5 ≤ BMI < 25**	34	31.5
**25 ≤ BMI < 30**	45	41.7
**BMI ≥ 30**	27	25.0
**Self-rated health status (N = 109)**		
**Excellent/very good**	42	38.5
**Good**	44	40.4
**Fair/poor**	23	21.1
**No. of chronic diseases (N = 111)**		
**0**	15	13.5
**1, 2**	56	50.5
**≥3**	40	36.0

The average BMI was 27.2 kg/m^2^, and 66.7% of participants were identified as overweight/obese, whereas 1.8% of them were underweight. More participants rated their health status as “good” (40.4%) than any other category and 21.1% rated their health unfavourably as either “fair” or “poor”. High blood pressure (62.2%) and arthritis or rheumatism (41.4%) were the most frequent diseases reported by the subjects. In this sample, only 15 subjects did not have any long-term conditions, while 86.5% of the participants reported one or more current illnesses. Long-term conditions included any of the following-asthma, osteoporosis, high blood pressure, chronic bronchitis or emphysema, diabetes, cancer, heart disease, depression, urinary disorders, stomach or intestinal ulcers, arthritis or rheumatism, and others that have lasted or are expected to last 6 months or more.

### Dietary intake

The average daily intakes of macro- and micro-nutrients according by gender are shown in Table 2. The mean energy intake of the study population was 2475.04 kcal per day, and the median value was 2352.68 kcal per day. Males presented a higher intake of total energy, all kinds of fat, and sodium, whereas females had a higher intake of other nutrients. Significant gender differences were only found in total energy, fat (total, saturated, and polyunsaturated fat), and sodium.

**Table 2:** Estimated overall and gender-specific dietary intakes of NL senior residents

**Nutrients**	**Total**			**Female (n = 69)**		**Male (n = 42)**		**p-value**^**a**^
	**Mean**	**SD**	**Median**	**Mean**	**SD**	**Mean**	**SD**	
**Energy (kcal)**	2475.04	855.21	2352.68	2472.31	745.33	2479.51	1020.22	.01*
**Protein (g)**	97.38	32.52	97.97	99.63	29.82	93.70	36.60	.30
**Carbohydrates (g)**	317.83	117.29	303.58	323.94	105.99	307.80	134.58	.06
**Dietary fibre (g)**	26.02	10.36	25.33	27.27	9.94	23.97	10.82	.55
**Fat (g)**	93.18	40.00	88.20	90.70	35.06	97.26	47.19	.01*
**Saturated (g)**	29.15	13.13	26.78	28.01	11.31	31.01	15.63	.03*
**Monounsaturated (g)**	34.17	15.39	32.21	33.79	14.57	34.81	16.82	.19
**Polyunsaturated (g)**	18.62	10.16	16.43	18.12	8.86	19.43	12.076	.00*
**Cholesterol (mg)**	301.36	135.70	282.02	301.77	140.02	300.67	129.95	.95
**Vitamin A (RAE)**^**b**^	1263.06	584.49	1203.69	1351.45	579.65	1117.84	569.70	.76
**Vitamin B6 (mg)**	2.23	.73	2.08	2.33	.72	2.06	.728	.67
**Vitamin B12 (mcg)**	7.07	3.95	6.03	7.46	4.02	6.43	3.80	.58
**Vitamin C (mg)**	222.93	130.38	202.00	240.63	135.75	193.86	116.83	.23
**Vitamin D (IU)**	340.68	188.62	296.30	344.78	171.81	333.95	215.46	.26
**Vitamin E (mg)**	8.59	3.92	7.99	9.02	4.01	7.89	3.69	.73
**Folate (DFE)**^**c**^	421.75	149.25	404.52	437.79	138.30	395.41	163.99	.22
**Vitamin K (mcg)**	136.14	90.06	115.68	149.39	100.84	114.38	64.15	.08
**Calcium (mg)**	1213.03	556.99	1145.18	1237.90	503.22	1172.18	640.00	.30
**Copper (mg)**	1.93	.81	1.80	2.01	.79	1.79	.84	.95
**Iron (mg)**	16.40	6.47	16.07	16.64	5.64	16.02	7.70	.08
**Magnesium (mg)**	379.09	141.91	363.80	391.67	127.97	358.42	161.75	.41
**Manganese (mg)**	4.99	2.06	4.68	5.13	1.82	4.76	2.40	.15
**Phosphorus (mg)**	1843.67	725.03	1812.13	1868.23	637.85	1803.34	856.07	.11
**Selenium (mcg)**	124.47	74.62	105.78	124.73	64.01	124.03	90.23	.59
**Sodium (mg)**	3738.58	1767.73	3424.19	3728.32	1598.64	3755.44	2035.86	.04*
**Zinc (mg)**	12.85	4.51	12.30	13.15	4.05	12.37	5.19	.15

^a^Statistical analysis using *t*-test *Significance of the difference by gender (p-value < 0.05).

^b^RAE, retinol activity equivalents.

^c^DFE = dietary folate equivalents, 1.0 DFE = 1.0 mcg food folate.

### Adequacy of dietary intakes

**Figure 1:**
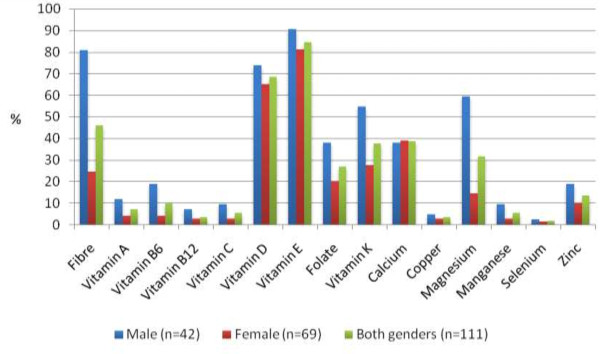
Estimated overall and gender specific prevalence of micronutrient intake inadequacy among senior residents from Newfoundland and Labrador.

The dietary intake adequacy for micro-nutrients and macronutrients are presented in Figure 1 and Table 3, respectively. Figure 1 shows the proportion of study subjects with micronutrient intakes below the appropriate EAR. The nutrients for which the largest proportion of subjects had inadequate intakes (less than the appropriate EAR) were vitamin E (84.7%), and vitamin D (68.5%). Many subjects appeared to be consuming less than recommended levels of vitamin E (90.5%), vitamin D (73.8%), and magnesium (59.5%). However, none of the participants had inadequate intakes of iron and phosphorus during the period under investigation. The results also suggest that a sizeable proportion of subjects have inadequate intakes for multiple micronutrients: 26% of subjects have inadequate intakes for over 5 nutrients, another 42% for 3–4 nutrients, and 25% for 1 or 2 nutrients.

**Table 3:** Number and proportion of study participants with AMDR in suggested ranges expressed as intake contribution to total dietary

**Participants n (% of sample)**	**Carbohydrate AMDR 45-65%**	**Protein AMDR 10-35%**	**Fat AMDR 20-35%**	**Polyunsaturated fat AMDR 5-10%**
**Total**	94(85.45%)	108(98.78%)	67(60.91%)	71(64.55%)
**Male**	36(85.71%)	42(100%)	26(61.90%)	26(61.90%)
**Female**	58(85.29%)	66(97.06%)	41(60.29%)	45(66.18%)

AMDR: Acceptable Macronutrient Distribution Ranges.

Table 3 shows the macronutrient (including carbohydrate, protein, fat and polyunsaturated fat) intake contributions to total energy according to AMDR. Taking into consideration with or without gender, more than 85% participants have adequate carbohydrate intake, over 97% of them take adequate protein, and greater than 60% of samples meet fat and polyunsaturated fat intake adequacy. However, an excessive fat intake was observed in 38.1% of males and 36.76% of females as their intakes exceeded the AMDR. Also 11.9% of males and 5.9% of females consumed polyunsaturated fats in excess of the AMDR.

Table 4 presents the different levels of intake according to RDAs. For both genders, over half of participants fell into the range of less than 66% of RDA for vitamin D and vitamin E. Over one quarter of the male participants was additionally below the 66% of RDA for dietary fibre and magnesium. Females were more likely to have adequate intakes in other nutrients (such as dietary fibre and folate). There was a great proportion of participants with sodium intake exceeding the tolerable upper intake level (UL: 2300 mg/day), 69.05% of males and 84.06% of females.

**Table 4:** Percentage of subjects into different levels of dietary intake according to RDAs for males (n = 42) and female (n = 69)

**Nutrients**	**Level of intake for males n (%)**					**Level of intake for females n (%)**				
	***Recommendation RDA***^***b***^	**<33% RDA**	**33%-65% RDA**	**66%-99% RDA**	**≥100% RDA**	***Recommendation RDA***^***b***^	**<33% RDA**	**33%-65% RDA**	**66%-99% RDA**	**≥100% RDA**
Dietary fibre (g)^a^	*30*	2(4.8%)	15(35.7%)	17(40.5%)	8(19.0%)	*21*	1(1.5%)	4(5.8%)	12(17.4%)	52(75.4%)
Vitamin A (RAE)	*900*	1(2.4%)	5(11.9%)	8(19.0%)	28(66.7%)	*700*	0	2(2.9%)	5(7.2%)	62(89.9%)
Vitamin B6 (mg)	*1.7*	0	3(7.1%)	11(26.2%)	28(66.7%)	*1.5*	0	2(2.9%)	3(4.3%)	64(92.8%)
Vitamin B12 (mcg)	*2.4*		1(2.4%)	3(7.1%)	38(90.5%)	*2.4*	0	1(1.5%)	2(2.9%)	66(95.6%)
Vitamin C (mg)	*90*	1(2.4%)	2(4.8%)	3(7.1%)	36(85.7%)	*75*	0	1(1.5%)	1(1.5%)	67(97.0%)
Vitamin D (IU)	*51-70y:600 > 70y:800*	12(28.6%)	20(47.6%)	8(19.0%)	2(4.8%)	*51-70y:600 > 70y:800*	25(36.2%)	29(42.0%)	13(18.9%)	2(2.9%)
Vitamin E (mg)	*15*	7(16.7%)	21(50.0%)	11(26.2%)	3(7.1%)	*15*	10(14.5%)	38(55.1%)	16(23.2%)	5(7.2%)
Folate (DFE)	*400*	1(2.4%)	8(19.0%)	15(35.7%)	18(42.9%)	*400*	0	4(5.8%)	25(36.2%)	40(58.0%)
Vitamin K (mcg)^a^	*120*	4(9.5%)	2(4.8%)	17(40.5%)	19(45.2%)	*90*	0	6(8.7%)	13(18.8%)	50(72.5%)
Calcium (mg)	*51-70y:1000 > 70y:1200*	2(4.8%)	7(16.7%)	11(26.2%)	22(52.4%)	*1200*	2(2.9%)	13(18.8%)	22(31.9%)	32(46.4%)
Copper (mg)	*0.9*	0	0	5(11.9%)	37(88.1%)	*0.9*	0	0	3(4.3%)	66(95.7%)
Iron (mg)	*8*	0	0	4(9.5%)	38(90.5%)	*8*	0	0	4(5.8%)	65(94.2%)
Magnesium (mg)	*420*	0	12(28.6%)	20(47.6%)	10(23.8%)	*320*	0	2(2.9%)	19(27.5%)	47(68.1%)
Manganese (mg)^a^	*2.3*	0	0	4(9.5%)	38(90.5%)	*1.8*	0	0	2(2.9%)	67(97.1%)
Phosphorus (mg)	*700*	0	0	2(4.8%)	40(95.2%)	*700*	0	0	1(1.5%)	68(98.5%)
Selenium (mcg)	*55*	0	0	3(7.1%)	39(92.9%)	*55*	0	1(1.5%)	3(4.3%)	65(94.2%)
Sodium (mg)^a^	*51-70y:1300 > 70y:1200*	0	0	2(4.8%)	40(95.2%)	*51-70y:1300 > 70y:1200*	0	0	1(1.4%)	68(98.6%)
Zinc (mg)	*11*	0	6(14.3%)	14(33.3%)	22(52.4%)	*8*	0	1(1.5%)	7(10.1%)	61(88.4%)

^a^adequate intake (AI) used.

^b^recommended dietary allowance (RDA).

According to results of logistic regression analysis (not shown), only age of more than 70 years was associated with vitamins D inadequacies (OR = 0.31, 95% CI 0.12-0.83, *p* < 0.05). Also, the association between health behaviour (alcohol use &physical activity) and chronic diseases was analyzed; however, there was no relationship.

## Discussion

Research on the adequacy of nutrient intakes has recognized the greater vulnerability of certain population subgroups, such as elderly, women, and blacks [20]. Dietary intake data on NL seniors is limited, the only two credible sources being the provincial survey conducted in 1996–1997, Nutrition Newfoundland and Labrador (NNL) [21], and the CCHS Cycle 2.2 of 2004 [8]. The present study is therefore an important contribution to the current literature on the dietary status of the elderly in NL. As for NNL and CCHS, the current study addressed intakes of nutrients from food only. It reports dietary intakes from food that are less than the recommended level for a number of nutrients (including vitamin E, vitamin D, calcium, and magnesium). This suggests that a significant proportion of noninstitutionlized seniors residing in NL did not meet daily nutrient requirements supported by Health Canada [18,22]. Inadequate intakes of vitamin D, calcium, vitamin A, and magnesium by senior Canadian adults were also noted by CCHS [8]. It is noteworthy that the prevalence of inadequate vitamin D intakes in seniors of NL exceeds 20%, which is lower than the corresponding number of Canadian adults reported in the literature and the reverse was true for calcium and vitamin A inadequacy [8].

An adequate dietary intake should contribute to an adequate nutritional status which in turn should support general health and successful aging. The supports to healthy aging could be associated with a healthy body composition and/or delay in onset or even prevention of such age-related disorders as depression and diabetes mellitus [23]. Nonetheless, inadequacy of dietary intake seems common in seniors in both developing and developed countries. Inadequate dietary intakes of energy, folate, vitamin D, vitamin B-6, calcium and zinc have been reported in elderly people over 60 years old [24-26]. For example, recent studies suggested that more than a half of elderly Americans (≥71 years) had inadequate intakes of vitamin A and E, and over one quarter of US seniors’ intakes of vitamin B12, C, D and K, folate were below EAR [27,28]. In a recent seven-year cohort study of elderly women residing in Australia, researchers found that intakes of vitamins and minerals all declined with age and subjects had suboptimal intakes of folate, vitamin E and calcium at all-time points. Similarly, Johnson *et.al* reported that elderly persons were consuming more than the recommended amount of protein, but the average intakes of many vitamins and minerals were less than optimal based on the average intakes [29].

According to the present results, inadequate intakes of vitamin D and vitamin E from food are common in NL seniors. This finding is supported by results of investigations into the elderly populations in many countries’ [30-32]. Deficiency of vitamin D and calcium have been linked to an increased incidence of osteoporosis [3]. Although dietary intakes of vitamin D were found to be less than recommended, much of the body’s needs for this nutrient have been shown to be supported by dermal production of the vitamin. Nevertheless, dermal production would be expected to be less in the elderly who normally spend only limited time in the sunlight [33]. This is of particular concern for NL residents because of the long winter and limited sunshine in NL. No clinical symptoms were apparent to suggest a late stage vitamin E deficiency in our respondents however a low consumption in the long term could be associated with hemolytic anemia and influence antioxidative activity [31,32]. Excessive intakes of fat and sodium predispose seniors to an increased risk of developing cardiovascular diseases [34], liver diseases [35], and other age-related diseases [36,37]. Roebothan [21] found total energy intake to be high in NL seniors and that more dietary energy was contributed from fat and less from carbohydrate than is recommended. Similar to our findings, she also reported inadequate fibre and mineral intakes in this population.

There is a growing appreciation for the value of a focus on the consumption of foods and food groups rather than a focus on single components of foods such as nutrients [38]. Although the authors support this transition they feel that the investigation of nutrient intakes by this particular population is far from complete. Future work with this group should consider intakes of foods and food groups and their patterns of consumption.

The current study is not without limitations. First, the sample size is relatively small and it offers limited statistical power. Due to limited data collected on human requirements for some micronutrients such as vitamin K, magnesium, and dietary fibre, appropriate EAR and RDA cannot be developed. In place of these dietary recommendations adequate intakes (AI), intakes of nutrients estimated to maintain health in healthy subgroups of the North American population, are used. The imprecision of AI values does not follow for the credible estimation of an adequate or inadequate dietary intake [39].

Another limitation is that this study did not account for nutrient intakes from supplements. Health Canada reported in 2005 that seven in ten Canadians had tried natural health products such as vitamins, and 38% of these used them daily [36]. However, our unpublished data has suggested that the use of supplements in NL is lower than that in other parts of Canada. Furthermore, the most recent census in NL [39] indicates that the female to male ratio (F:M) is around 1.2:1 for the target age range, while the F:M ratio of our respondents was 1.6:1. Lastly, due to a relatively small sample size, we were not able to separately assess individuals aged 80 years and older. We believe that the nutritional status of seniors over 80 years should receive more attention in future studies. We intend to suggest future researches could focus on this point further.

## Conclusion

In conclusion, our study suggests that the dietary intakes of NL seniors appear to be inadequate in many respects, vitamins D and E in particular. The inadequacy seems to be pronounced in males.

## Abbreviations

NL: Newfoundland and Labrador; BMI: Body mass index; CI: Confidence interval; OR: Odds ratio; SD: Standard deviation; ESHA: Elizabeth Stewart Hands and Associations; NNL: Nutrition Newfoundland and Labrador; CCHS: Canadian Community Health Survey; USDA: United State Department of Agriculture; IOTF: International Obesity Task Force; FFQ: Food Frequency Questionnaire; GHQ: General Health Questionnaire; DRI: Dietary Reference Intake; EAR: Estimated Average Requirement; AI: Adequate intake; AMDR: Acceptable Macronutrient Distribution Ranges; RDA: Recommended daily allowance; SPSS: Statistical Package for Social Science.

## Competing interests

The authors of this paper indicated no competing interest.

## Authors’ contributions

PW contributed to the conception and design of this manuscript. JY and LL analyzed the data and drafted the first version of the manuscript. PPW, BR, AR, ZC, YY subsequently revised the manuscript. JY, LL and ZC were responsible for the data collection and had full access to the data. All the authors provided final approval.

## Authors’ information

PW is a professor of epidemiology, BR is a professor of dietetics/nutrition, and YY is an assistant professor of biostatistics. RY is the manager of Health Research Unit. They are faculties of Memorial University of Newfoundland (MUN). LL and ZC are current Master’s students in MUN, while JY is a lecturer in Tianjin Medical University, who was working with others one year as a visiting scholar.

## Pre-publication history

The pre-publication history for this paper can be accessed here:

http://www.biomedcentral.com/1471-2458/14/302/prepub
